# Social Hygiene

**Published:** 1911-01

**Authors:** 


### EDITORIAL DEPARTMENT.

### SOCIAL HYGIENE.

The movement for "Social Hygiene," which is only another name for "public health," or, rather, sanitation, is now worldwide. The aim is principally the prevention of the spread of venereal disease, and other things which strike at the very foundation of our social structure, threatening, indeed, the integrity of the human race; and the method is—education. At last, the medical profession seems to be awakening to a realization of the fundamental truth that the rational method of dealing with disease, and, in fact, with all evils, is prevention and not cure, and a crusade has been started looking to the eradication, so far as may be possible, of preventable diseases. It is a reproach to our civilization that while precautions more or less effective are instituted by law against epidemic infections of all other kind, no notice whatever is taken of that worst of all epidemic infections,—the one that is most insidious,—because hidden, and most far-reaching in its deadly effects upon the human family—the venereal infection. How these diseases, originating in that fixed feature of all "civilizations"—prostitution,—are conveyed to the innocent—the wives, and mothers of the children—upon whose health and welfare and integrity depend that of future generations, is a familiar story to the medical profession, but the people know nothing of it, and heretofore a kind of false modesty—an absurd shamefacedness—has withheld from the boys and girls who will become the fathers and mothers of the next generation all knowledge of the physiology of sex and of procreation. There is an awakening, and our sons and daughters should be enlightened on those vital subjects which so nearly concern not only themselves but all posterity. But—the how? That is one of the purposes of the world-wide movement referred to. There is a society for social hygiene in most of the States, integers of a national society, organized, I believe, through the efforts of the pioneer—Dr. Prince A. Morrow, of New York. The subject of venereal diseases is only one of the many which are receiving attention. The extensive self-drugging of the people by villainous patent medicines—habit-forming drugs; the evil of alcohol—which many of these concoctions contain, dram drinking, the infernal saloon.

Ah! well, the hope of reform seems Utopian—because of the many evils and because of the many and insurmountable obstacles. Prostitution can never be eradicated. It is inherent in our social system, and no civilization has ever been able to deal effectively with it, and hence one difficulty in dealing with one of its results—the spread of the venereal infection. And as to patent medicines—the manufacturers have the ear of the credulous public through the subsidized newspapers whose income is thus derived, and their lies go uncontradicted; those pages are shut to reformers—because there is no money to pay for space. And as to "regulating matrimony"—pooh! The corruptibility of men—and of some "doctors," and the corrupting power of money—constitute insurmountable difficulties. Don't you know that if the law required a "clean bill of health" as a condition to marriage that there are doctors in Texas and everywhere who would *sell* them? We know of certain medical examining boards who made a business of selling license to practice, and we read of a doctor who owns a drug store who prescribed whisky for a fellow and filled the prescription himself.

The meeting of the Texas State Society of Hygiene will be held in Austin this month—January, 1911—and an attractive program has been arranged. Certain things have been agreed upon in advance, and which the incoming Legislature (January 17th) will be asked to consider. The President of the Society is Dr. Malone Duggan, of San Antonio; Dr. Daniel, of Austin, is Vice-President, and Dr. T. Y. Hull, of San Antonio is Secretary. (See program.) There will be other speakers, and the discussion will be general, many women of note being expected to take part. The time has come when the people should take their fingers from before their eyes and call a spade a spade, and hear it called a spade. In popular education alone lies any hope of any reform in the evils that beset society and threaten the integrity of the human race.

---

### The Texas State Society of Social Hygiene,

### SAN ANTONIO, TEXAS.

*Officers*—Dr. Malone Duggan, President, San Antonio; Dr. F. E. Daniel, First Vice-President, Austin; Prof. A. Caswell Ellis, Second Vice-President, Austin; Mrs. Eli Hertzberg, Third Vice-President, San Antonio; Dr. Theo. Y. Hull, Secretary, San Antonio; Mr. W. L. Evans, Treasurer, San Antonio.

*Executive Committee*—Dr. Malone Duggan, Chairman; Dr.

Theo. Y. Hull, Secretary; Dr. W. F. McCaleb, Hon. C. H. Jenkins, Rev. A. W. S. Garden, Hon. J. G. Willacy, Mr. W. L. Evans, Dr. Frank D. Boyd, Dr. J. R. Nichols, Dr. J. M. McCutchan, Dr. W. A. King, Dr. J. W. Torbett, Dr. F. C. Walsh, Dr. Joe Reuss, Dr. Frank Paschal.

Program of the Meeting of the State Society of Social Hygiene to be held January 26 and 27, 1911, Austin, Texas.

First Session—January 26th, 8 p. m.

Opening Address, Dr. F. E. Daniel, Austin.

"The Blackest Stain on Our National Greatness—the Ignorance of the Nature of Sex and the Devastation of the Home by the Social Plague," Dr. John S. Turner, Dallas.

"Should Sex Hygiene Be Taught in the Home and School?" President S. E. Mezes, University of Texas, Austin.

Second Session—January 27th, 8 p. m.

"Some of the Causes of National Deterioration and Degeneracy," Dr. Holman Taylor, Editor *State Journal of Medicine,* Fort Worth.

"The Chief Function of the State to Develop a Normal Race," Judge R. L. Batts, Austin.

The introduction of resolutions.

